# Core-shell structured titanium-nitrogen alloys with high strength, high thermal stability and good plasticity

**DOI:** 10.1038/srep40039

**Published:** 2017-01-06

**Authors:** Y. S. Zhang, Y. H. Zhao, W. Zhang, J. W. Lu, J. J. Hu, W. T. Huo, P. X. Zhang

**Affiliations:** 1 Northwest Institute for Nonferrous Metal Research, Xi’an Shanxi 710016, China; 2 Nano Structural Materials Center, School of Materials Science and Engineering, Nanjing University of Science and Technology, Nanjing 210094, China

## Abstract

Multifunctional materials with more than two good properties are widely required in modern industries. However, some properties are often trade-off with each other by single microstructural designation. For example, nanostructured materials have high strength, but low ductility and thermal stability. Here by means of spark plasma sintering (SPS) of nitrided Ti particles, we synthesized bulk core-shell structured Ti alloys with isolated soft coarse-grained Ti cores and hard Ti-N solid solution shells. The core-shell Ti alloys exhibit a high yield strength (~1.4 GPa) comparable to that of nanostructured states and high thermal stability (over 1100 °C, 0.71 of melting temperature), contributed by the hard Ti-N shells, as well as a good plasticity (fracture plasticity of 12%) due to the soft Ti cores. Our results demonstrate that this core-shell structure offers a design pathway towards an advanced material with enhancing strength-plasticity-thermal stability synergy.

With the high-speed development of modern industries, materials are often required to simultaneously possess more than two excellent properties, which are called multifunctional materials^1^,^2^. For example, materials for structural applications are necessary to have high strength and good ductility as well as good thermal stability. Conductive wires used for high-speed trains need to possess both high conductivity and high wear-resistance ability. However, some properties are often trade-off with each other, or seldom co-existing simultaneously. For instance, strength and plasticity are consuming each other: enhancing strength will sacrifice plasticity, and recovering plasticity has to lose strength. The extensively investigated nanostructrued (NS) and ultrafine grained (UFG) materials in past three decades could possess more than 10 times higher yield strength than their coarse-grained (CG) counterparts, but disappointingly low ductility (<10%)^3^,^4^,^5^,^6^,^7^,^8^,^9^. Moreover, the strength and thermal stability of NS and UFG materials are also mutually exclusive: their grain growth temperatures are much less than 0.5 *T*_*m*_ (*T*_*m*_ is melting point)^10^. The underlying determined mechanism for strength-plasticity dilemma is dislocation dominated plastic deformation. Refining the CG down to NS and UFG regions takes away the space for dislocation accumulation and multiplication, therefore lowers the plasticity which is related with dislocation nucleation and slip. The low thermal stability of bulk NS materials is because of high total grain boundary (GB) enthalpy due to high GB volume fraction, which simultaneously acts as a driving force for GB migration and dislocation slip barriers for high strength.

To design multifunctional materials with good trade-off properties or break the property paradox, one need tailor different microstructures or mechanisms for different trade-off properties, respectively. For instance, both high strength and high ductility are achieved in ultrafine grained (UFG) transformation-induced-plasticity (TRIP) steel where high strength is from grain refinement and high ductility from phase transformation^11^. More examples, in bimodal Cu, the UFG grains contribute to high strength and CGs to high ductility^6^. In gradient nano-grained Cu, the nano-grained surface has high wear-resistance ability, and the CG core has high plasticity^12^,^13^. A composite structure was also used for designing Ti alloy to obtain both high strength and plasticity^14^. For strength-thermal stability paradox, both kinetic (reducing GB mobility by second phase drag or solute drag) and thermodynamic (GB segregation, low-energy GBs) strategies were proposed to stabilize nanostructures up to 0.7*T*_*m*_^15^,^16^.

In this work, we designed and synthesized bulk core-shell structured Ti-N alloys by mean of spark plasma sintering (SPS) of nitrided Ti particles. The bulk core-shell Ti-N alloys, consisting of isolated soft CG Ti cores and continuous hard Ti-N solid solution shells, exhibit high yield strength of 1.4 GPa and high thermal stability over 1100 °C (0.7 *T*_*m*_) as well as a good plasticity (fracture plasticity of 12%). In addition, the thickness and distribution of the hard Ti-N shells can be optimized by tailoring preparation parameters.

## Results and Discussion

### Powders characterization

**Figure 1:**
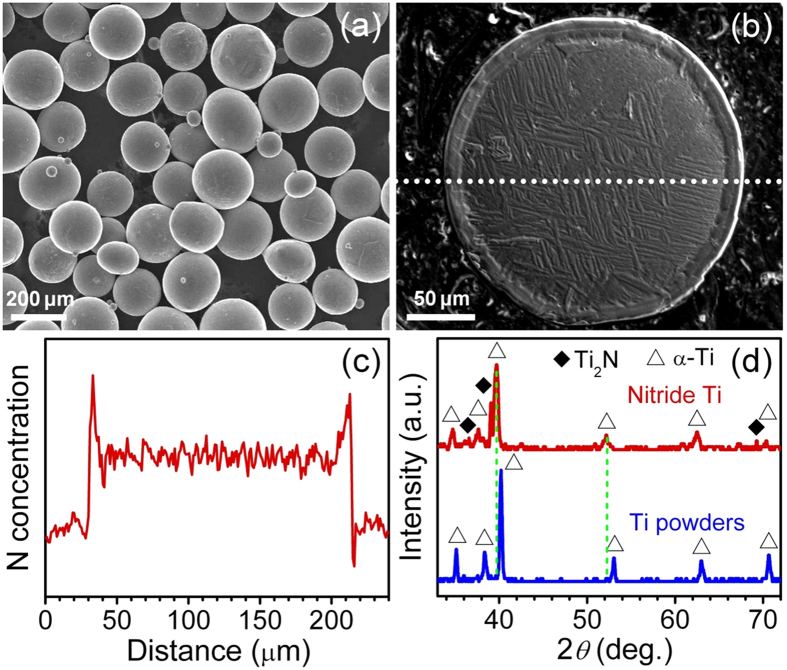
Characterization of the powders. (**a**) SEM image of the initial Ti powders. (**b**) EP micrograph of the cross-sectional nitrided Ti powder. (**c**) EP analysis of N concentration along the white dotted line in (**b**). (**d**) XRD patterns of the nitrided and initial Ti powders.

The initial commercial pure (CP) Ti powders with 0.08 wt.% O and 0.02 wt.% N were imaged by scanning electron microscopy (SEM), as shown in Fig. 1a. The particle size is ranging from 100 to 250 μm. After nitriding at 1000 °C for 15 min in N atmosphere, metallographic analysis, as shown in Fig. 1b, revealed that a core-shell structure with a shell thickness of 15 μm was formed. The lamellar α-Ti in the inner core was nucleated from β-Ti and preferred to grow along the direction with the lowest strain energy based on the theory of solid phase transformation when cooling from the temperature above α-β transus point (882 °C)^17^. An electro probe (EP) compositional profile (Fig. 1c) reveals that the outer shell is enriched in N and the N concentration decreases gradually from the shell surface to the core-shell interface. X-ray diffraction (XRD) analysis (Fig. 1d) shows that the initial Ti powders are α-Ti, and after nitriding, besides α-Ti, major Ti_2_N peaks are resolved with a volume fraction of about 4%^18^. Based on the calculated XRD penetration depth in pure Ti, i.e. 11 μm^19^, the Ti_2_N layer thickness can be estimated as about 0.44 μm, and the α-Ti peaks are also from the shell. Therefore, the shell is mainly composed of an outmost surface Ti_2_N layer and an α-Ti layer enriched N solution. This could be further verified by the fact that the α-Ti peaks of the nitrided Ti powders shift towards lower diffraction angles compared with those of the CP-Ti, which was resulted from Ti lattice expansions by the interstitial N atoms.

### Compact characterization

**Figure 2:**
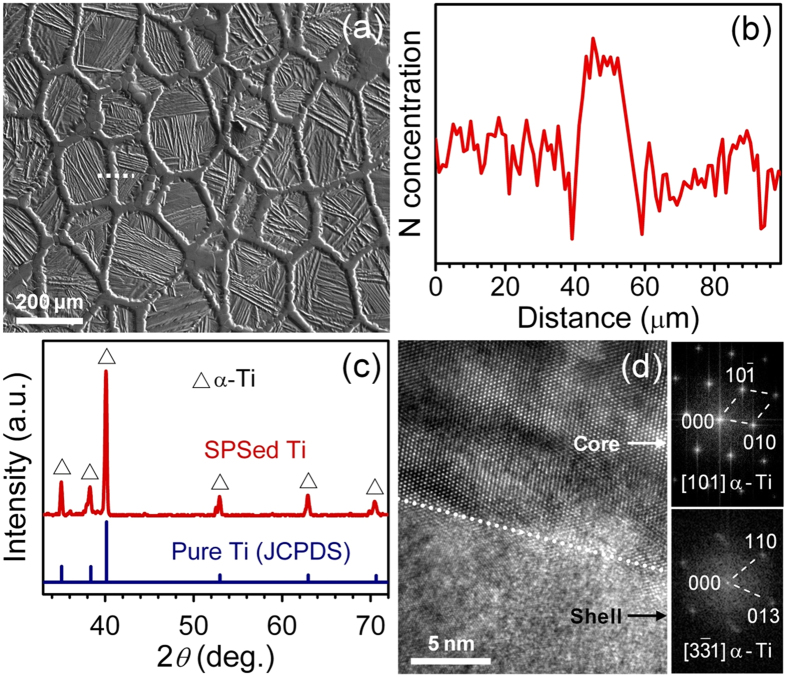
Characterization of the compact. (**a**) EP micrograph of the SPSed Ti-N alloys. (**b**) EP analysis of N concentration along the white dotted line in (**a**). (**c**) XRD patterns of the SPSed Ti-N alloys. (**d**) High-resolution TEM image and the corresponding Fourier transform (FFT) patterns of the SPSed Ti-N alloys, both core and shell are α-Ti and have obvious interface.

The SPS sintering process at 1100 °C fabricated fully dense specimens with a density of 4.52 g/cm^3^ (close to the theoretical value of CP Ti: 4.51–4.53 g/cm^3^) and kept the initial core-shell structure: polygon cores due to plastic flow during sintering were separated by continuous shell networks (Fig. 2a). The polygon cores are still lamellar α-Ti and have sizes comparable with those of initial cores. The shell network thickness ranges from about 30 to 50 μm, larger than that of nitride particles. Composition and XRD analyses (Fig. 2b,c) revealed the shell network is only α-Ti with higher N concentration relative to the core. Therefore, the Ti_2_N surface layer in the nitride shell was transformed to Ti-N solid solution during SPS sintering. The above results were further verified by high resolution transmission electron microscopy (HREM) observation in Fig. 2d. Both core and shell are α-Ti and have obvious interface between them.

### Formation mechanism

Conventional powder metallurgy (PM) sintering is difficult to prepare the core-shell structure because of rapid grain growth and sufficient solute diffusion during sintering. The present SPS process consolidates powders in a short time and under a high pressure which impedes the grain growth and solute diffusion^20^. However, our previous work indicated that SPS of nitrided Ta particles only prepared a gradient N concentration without core-shell structure^21^. During SPS of Ta, current and Joule heat are focused at the neck between the particles, resulting in a temperature gradient from particle center to surface^22^, this temperature gradient confined the N diffusion and resulted in the gradient N structure.

**Figure 3:**
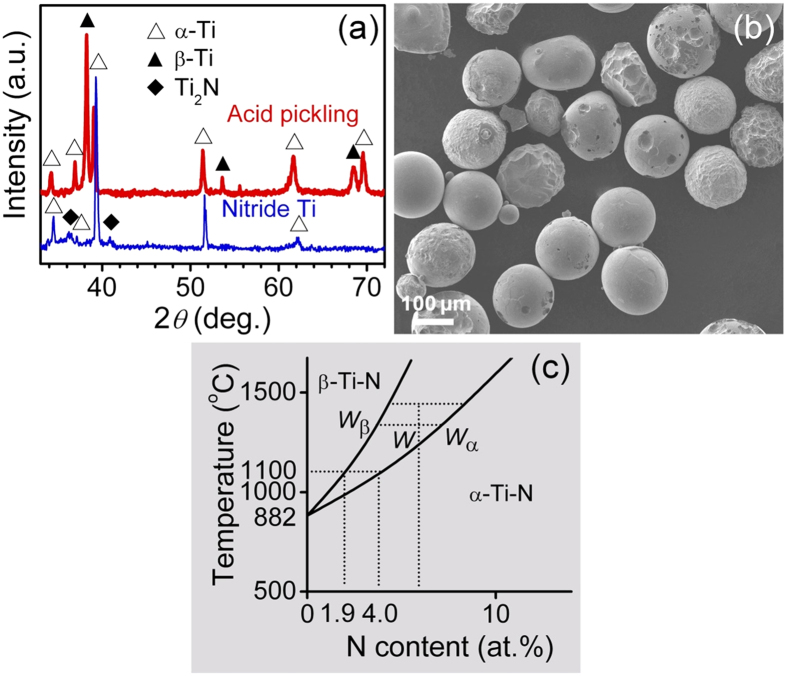
High temperature XRD results and the partial Ti-N phase diagram. (**a**) XRD patterns of the nitrided powders at 1100 °C with and without acid pickling. (**b**) SEM image of the acid pickled nitrided Ti powders, the Ti_2_N surface layer and part of the Ti-N solution layer in the shell were removed. (**c**) Schematic representation of the partial Ti-N phase diagram^23^.

To explore the Ti-N core-shell formation mechanisms, we further carried out XRD analyses at sintering temperature (1100 °C) on the nitrided Ti particles with and without acid pickling, as shown in Fig. 3a. By acid pickling, the Ti_2_N layer and part of the Ti-N solution layer in the shell were removed, as shown in Fig. 3b, and the core has chance to be involved in the diffraction. The XRD pattern of the nitrided powders at 1100 °C without acid pickling did not show obvious change compared with the XRD pattern obtained at room temperature, indicating that the α-Ti-N and Ti_2_N in the shell did not change to β-Ti at 1100 °C. While after acid pickling, Ti_2_N peak disappears and both α- and β-Ti peaks showed up indicating the α-Ti core was transformed to β-Ti at 1100 °C. From Ti-N phase diagram in Fig. 3c 23. One can see N solute could stabilize α-Ti and move the α-β transus point to higher temperatures. At 1100 °C, α-Ti-N solution with >4 at.% N is stable, that with <1.9 at.% N will transform into β-Ti, and those between 1.9 and 4 at.% N will separate into both α- and β-Ti. Combined with the above XRD results, one could deduce that the N concentration in the shell is larger than 4 at.%, and that in the core is smaller than 1.9 at.%.

**Figure 4:**
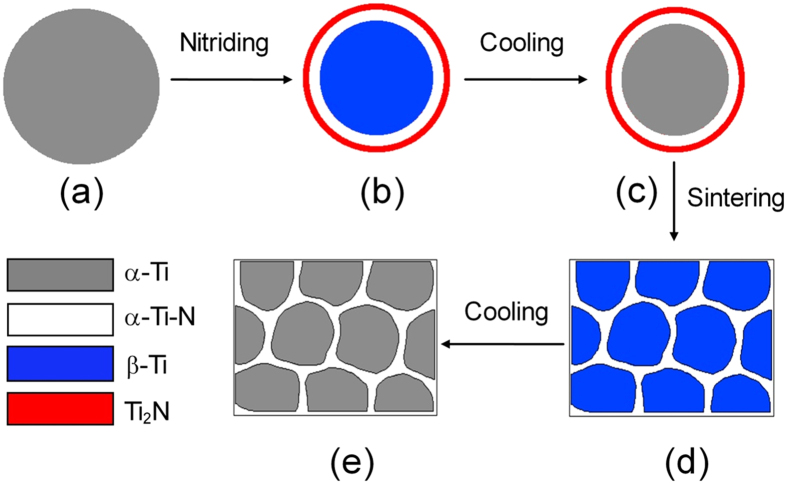
Schematic illustration. A schematic illustration depicting the formation procedures of the core-shell Ti-N alloys during nitriding (**a**–**c**) and SPS sintering (**c**–**e**) processes.

The above core-shell structure formation can be presented schematically in Fig. 4. During nitriding at 1000 °C, the N diffusion results in the formation of the outmost Ti_2_N surface layer (red ring) and the α-Ti-N solution sub-layer (white ring) in the shell. The core is β-Ti (blue circle) transformed from α-Ti. When cooling down to room temperature, the β-Ti core was transformed back into α-Ti resulting in a core-shell structure (Fig. 4c). When sintering at 1100 °C, the N diffusion of the outmost Ti_2_N surface layer leads to its disappearance (Fig. 4d), while the α-Ti-N solution layer still remained its structure due to high N concentration. Finally, the β-α transformation of the β-Ti core occurred after cooling down to room temperature (Fig. 4e). Therefore, the formation of the present core-shell structure can be attributed to occurrence of α and β phase transformations, in contrast to the gradient N structure in Ta which has not experienced phase transformations^21^.

### Thermal stability

**Figure 5:**
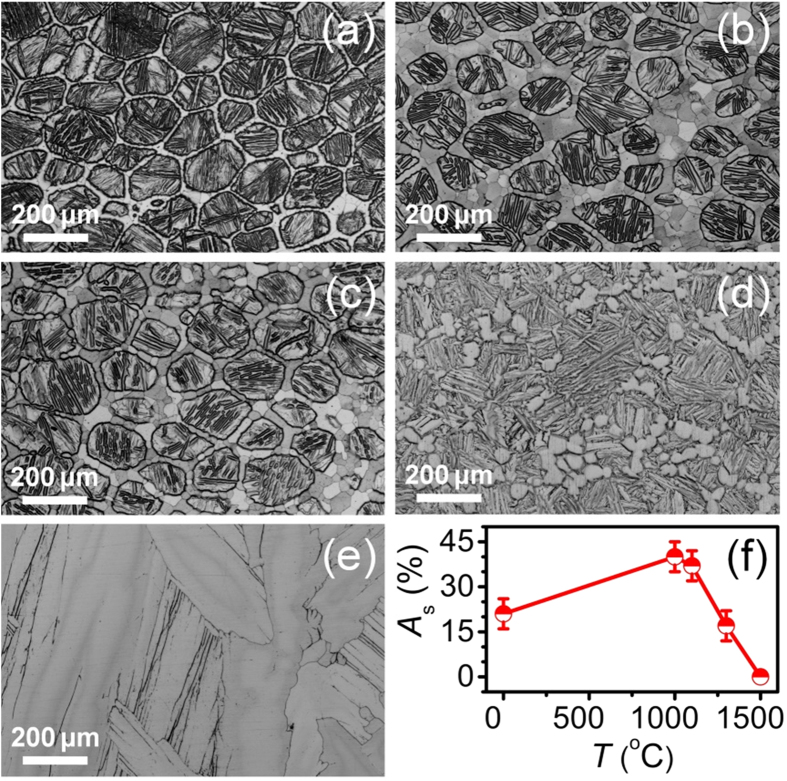
Microstructure evolution during annealing. Optical images of the SPS sintered specimen (**a**) and those annealed at 1000 (**b**), 1100 (**c**), 1300 (**d**) and 1500 °C (**e**) for 1 h, respectively. (**f**) Variations of shell area fraction *A*_*S*_ versus annealing temperature *T*.

To reveal the thermal stability of the core-shell structured Ti-N alloys, we performed annealing treatment at 1000, 1100, 1300 and 1500 °C for 1 h in a vacuum of 1 × 10^−3^ Pa, respectively. Figure 5 shows the optical micrographic images. Compared with the as-sintered Ti-N specimen (Fig. 5a), the Ti-N alloys showed a substantial increase in the shell thickness after annealing at 1000 °C, and the shell area fraction *A*_*S*_ increased from 21% to 40% (Fig. 5b). With further increasing annealing temperature *T* to 1100 °C, *A*_*S*_ decreased to 37% (Fig. 5c). The Ti-N shell tends to be non-continuous when annealed at 1300 °C (Fig. 5d) and disappears completely at 1500 °C where a homogeneous coarsened lamellar structure was formed (Fig. 5e). Prolonging annealing time did not lead to microstructural change, indicating that the core-shell Ti-N specimen approaches a stable state after 1 h annealing.

The above core and shell morphology changes during annealing can be understood on the basis of the Ti-N phase diagram in Fig. 3c 23. In term of the lever rule, the weight percentage of α-Ti shell network (*V*_*α*_) can be defined as:


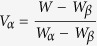


where *W, W*_*β*_, and *W*_*α*_ represent the N atomic percents in Ti alloy, β-Ti and α-Ti, respectively. With an increase in temperature, *W* − *W*_*β*_ is reduced significantly, while *W*_*α*_ − *W*_*β*_ is increased evidently, resulting in a dramatic reduction in *V*_*α*_. So it is reasonable to observe a decreased *A*_*S*_ with increasing *T*. The short time of SPS sintering process caused an incomplete N diffusion in the as-sintered Ti-N alloys, and subsequent annealing at 1000 °C for 1 h enhanced the N diffusion and therefore increased the shell thickness.

### Mechanical behaviors

**Figure 6:**
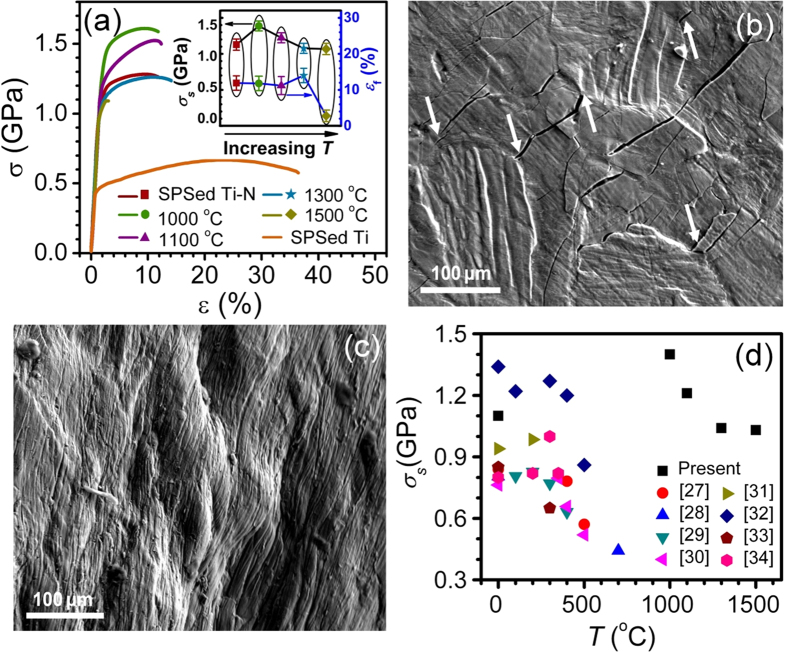
Mechanical behaviors. (**a**) Typical compressive true stress–strain curves of the SPSed CP-Ti, Ti-N alloy and annealed Ti-N specimens, inset shows the *T* dependences of the yield strength *σ*_*s*_ and fracture plasticity *ε*_*f*_. (**b**) and (**c**) Cross-sectional SEM images of the compressed SPSed + 1000 °C Ti-N alloys and CP-Ti, respectively. (**d**) *σ*_*s*_ versus *T* of the NS and UFG Ti as well as our results.

Compressive tests indicate that the SPSed CP-Ti has yield strength σ_s_ of about 400 MPa, as shown in Fig. 6a. The SPSed core-shell Ti-N alloy has enhanced σ_s_ of 1.1 GPa with moderate fracture plasticity *ε*_*f*_ of 12%. Moreover, there is strain hardening phenomenon after yielding: the flow stress increased from 1.1 to 1.3 GPa. After annealing at 1000 °C, the σ_s_ was further increased to 1.4 GPa, and the *ε*_*f*_ was still remained at 12%. With further increasing *T* up to 1300 °C, the Ti-N alloys exhibit reduced σ_s_ and unchanged *ε*_*f*_. The Ti-N alloy annealed at 1500 °C has significantly reduced *ε*_*f*_ of 2.8% accompanied with σ_s_ of 0.9 GPa.

The good relationship between σ_s_ and *A*_*S*_ indicates the evident strengthening effect of the Ti-N shell, which can be further explained in terms of the Hashin Shtrikman (H-S) theorem^24^: a hard phase encapsulates a soft phase will form the highest strengthening effect. In details, the strengthening effect of the Ti-N shell includes two parts. The first part is originated from the high strength of the Ti-N shell itself, which is caused by the high interstitial N concentration^25^. The second part is from the deformation limit of the Ti-N shell to the soft Ti cores. As shown in Fig. 6b, during compressive deformation, shear bands were confined within the individual soft Ti cores by the Ti-N shell, while the shear bands can extend to different grains in CP-Ti (Fig. 6c). Moreover, it is noted in Fig. 6b that a great number of micro-cracks are distributed in the Ti-N shells, suggesting that cracks were nucleated first within the Ti-N shell regions, and were blunted or impeded by the soft Ti cores (indicated by arrows), resulting in the moderate fracture plasticity. The mechanical performances are comparable to the Ti-base nanostructure-dendrite composites, which exhibited a compression *σ*_*s*_ of 1.31 GPa and *ε*_*f*_ of 14.5%^14^. In literature, several investigations have shown that high compression *σ*_*s*_ of 1.35 GPa and *ε*_*f*_ approaching 10% were obtained in a CP-Ti with 1.34 wt.% O and 0.3 wt.% N, which was believed to stem from non-equilibrium GB configuration and the bimodal grain structure^26^.

Figure 6d summarizes experimental data of σ_s_ versus *T* for NS or UFG CP-Ti in literatures^27^,^28^,^29^,^30^,^31^,^32^,^33^,^34^. The present core-shell Ti-N alloys possess much higher room temperature yield strength (>1 GPa) after high annealing temperatures *T* (>1000 °C) compared with the literature data in which σ_s_ of 1 GPa could be obtained only when *T* is smaller than 300 °C. When *T* > 300 °C, the σ_s_ in literature decreased dramatically below 450 MPa. In ref. 28, the annealing duration of 30 s at 700 °C resulted in the complete disappearance of the UFG structure. In the present work, further prolonging annealing at 1000 °C for 10 h did not change the morphologies of the Ti-N alloys, indicating their potential applications at high temperature above 1000 °C.

A core-shell structured bulk Ti-N alloy with soft Ti cores and Ti-N shells was successfully synthesized by means of a unique methodology combining nitriding of Ti powders and subsequent SPS sintering. Both phase transformation and SPS technology play key roles in obtaining such a novel architecture. The compacts exhibit substantially enhanced yield strength of 1.4 GPa together with apparent plasticity of 12% as well as high thermal stability of 1100 °C. Our results demonstrate that this novel architecture offers a designation pathway towards an advanced material with enhancing strength-plasticity-thermal stability synergy.

## Methods

### Specimen preparation

CP-Ti powders were chosen as the feedstock. Before nitriding process, the Ti powders were first encapsulated in a porous stainless steel mould which could rotate during nitriding to make a homogeneous reaction. Ti powders in the steel mould were then put in a tube furnace and nitrided at 1000 °C for 15 min in N atmosphere, and were finally cooled to room temperature. The consolidation of the Ti nitrided powders was carried out by using a SPS machine (Elenix company, Japan) in a vacuum of 10^−1^ Pa. The Ti nitrided powders were first packed into a cylindrical graphite die with an inner diameter of 30 mm. By controlling the electric current at a range of 2000 to 3000 amperes and a voltage range of 5 to10 volts, the compact powders were then heated up to 1100 °C at a heating rate of about 150 °C/min under a pressure of 10 MPa, and then were kept at 1100 °C under a pressure of 40 MPa for 5 min to obtain fully compacted specimens. After sintering, the load was removed, and the specimen was furnace cooled to room temperature.

### Characterization techniques

The density of the consolidated specimens is measured by the Archimedes method. The contents of O and N were analyzed by using a carrier gas hot extraction (O/N analyzer/Leco). To perform metallographic analysis, the hot-mounted nitrided Ti powders and the sintered specimens were mechanically polished via a standard metallographic procedure and subsequently etched using a solution of HF:HNO_3_:H_2_O = 1:1:3 in volume. The composition and surface morphologies were observed using a JEOL-JSM6460 scanning electron microscope (SEM) and a JXA-8230 electro probe (EP). XRD analyses were carried out using Cu K_α_ radiation with a Rigaku RU-300 at room temperature and an X’Pert Prox X-ray diffractometer at 1100 °C under Ar atmosphere, respectively. For acid pickling, the nitrided Ti powders were immersed in the a dilute solution of HF:H_2_O = 2:1 in volume for 1 h. HREM was performed by using a JEOL 2010 operated at a voltage of 200 kV. Thin foil samples for TEM observations were cut from the samples and thinned by ion thinning at low temperatures.

### Compression testing

Quasi-static uni-axial compression test was performed at room temperature with a strain rate of 5 × 10^−4^ s^−1^ by using a conventional CMT5105 mechanical testing system. Compression samples with sizes of 3 mm in diameter and 6 mm in length were cut from the sintered specimens according to ASTM (American society for testing and materials) standards.

## Additional Information

**How to cite this article**: Zhang, Y. S. *et al*. Core-shell structured titanium-nitrogen alloys with high strength, high thermal stability and good plasticity. *Sci. Rep.* **7**, 40039; doi: 10.1038/srep40039 (2017).

**Publisher's note:** Springer Nature remains neutral with regard to jurisdictional claims in published maps and institutional affiliations.
